# Phenolic Modified Ceramic Coating on Biodegradable Mg Alloy: The Improved Corrosion Resistance and Osteoblast-Like Cell Activity

**DOI:** 10.3390/ma10070696

**Published:** 2017-06-25

**Authors:** Hung-Pang Lee, Da-Jun Lin, Ming-Long Yeh

**Affiliations:** 1 Department of Biomedical Engineering, National Cheng Kung University, Tainan 701, Taiwan; qer6322129@gmail.com; 2 Department of Materials Science and Engineering, National Cheng Kung University, Tainan 701, Taiwan; larrylin111@hotmail.com; 3 Medical Device Innovation Center, National Cheng Kung University, Tainan 701, Taiwan

**Keywords:** Mg alloy, ceramics coating, molecular grafting, biocompatibility

## Abstract

Magnesium alloys have great potential for developing orthopedic implants due to their biodegradability and mechanical properties, but the rapid corrosion rate of the currently-available alloys limits their clinical applications. To increase the corrosion resistance of the substrate, a protective ceramic coating is constructed by a micro-arc oxidation (MAO) process on ZK60 magnesium alloy. The porous ceramic coating is mainly composed of magnesium oxide and magnesium silicate, and the results from cell cultures show it can stimulate osteoblastic cell growth and proliferation. Moreover, gallic acid, a phenolic compound, was successfully introduced onto the MAO coating by grafting on hydrated oxide and chelating with magnesium ions. The gallic acid and rough surface of MAO altered the cell attachment behavior, making it difficult for fibroblasts to adhere to the MAO coating. The viability tests showed that gallic acid could suppress fibroblast growth and stimulate osteoblastic cell proliferation. Overall, the porous MAO coating combined with gallic acid offered a novel strategy for increasing osteocompatibility.

## 1. Introduction

Magnesium and its alloys are considered as the next-generation biomaterials for tissue repair and reconstruction [1,2,3]. Magnesium alloys have comparable mechanical properties with natural bone, such as density, strength, and elastic modulus. Moreover, magnesium is also one of the main ions of bone tissue. Moreover, recent research shows that magnesium ions can induce cellular adhesion and bone formation, which are important functions for building strong bone-implant interfaces [4,5]. In this respect, magnesium alloys are suitable candidates for bone grafting, due to their osteoinductive and osteoconductive effects [6].

In order to successfully apply magnesium alloys as biodegradable metal implants, controlling the degradation rate in the physiological environment is the main aim of surface modification. Suitable corrosion behavior leads to good biocompatibility, sufficient mechanical properties, and a supportive structure for healing tissue. However, the high corrosion rate of magnesium in the presence of a physiological environment containing chloride ions limits its clinical application. The rapid corrosion behavior of acute surges in the pH value and concentrations of metallic ions may lead to the failure of surgery [7]. In addition, the corrosion reaction that occurs on the surface will produce hydrogen bubbles that obstruct the initial cell adhesion [8]. These adverse effects highlight the need to reduce the initial degradation rate and enhance its biocompatibility. Surface modification of magnesium is one of the effective ways to improve the corrosion behavior. To date, numerous surface modifications have been employed on biodegradable magnesium alloys, such as alkaline heat treatment [9], chemical conversion [10], electrochemical deposition [11], and micro-arc oxidation (MAO) [12,13]. All those methods provide better corrosion resistance with a lower degradation rate. However, the fragile binding force of these coatings undermine their clinical application, while MAO promises good performance with regard to practical use.

MAO can create an oxidized ceramic coating as a barrier on the metallic substrate to enhance corrosion resistance. Several previous papers indicate that the coating can increase cell and tissue compatibility [13,14]. The composition of the coating, such as forsterite, can stimulate the adhesion and proliferation of osteoblasts on the implants [14]. Introducing functional compounds on the interface can exert and induce some specific biological responses. However, in most cases, studies that aim at the immobilization of further molecules onto the MAO ceramic coating mainly focus on forming a hydrophobic layer or sealing pores to increase corrosion resistance [15,16]. Gallic acid (3,4,5-triphydroxyl-benzoic acid, GA), a phenolic acid, is abundant in green tea and red wine [17], and in recent years has drawn much attention due to its anti-tumoral, anti-bacterial, antioxidant, and anti-inflammatory properties [18,19]. In addition, it also shows selective regulation toward several cell lines, such as vascular endothelia and vascular smooth muscle cells [20]. Moreover, some reports imply that oxidative stress will hinder bone healing and osteogenesis, so that GA may have the potential to improve osteoblast’s function by reducing ROS (reactive oxygen species) [21].

In this study, Mg-6Zn-0.5Zr alloy in the form of ZK60 (Zn 5.3 wt %, Zr 0.3 wt %, and balance Mg) was used as the metallic substrate, as it is a good candidate for medical applications with good biocompatibility [22,23]. The main alloying elements in ZK60 are zinc, participating the bone formation process, and zirconium, a trace element in our bodies. However, the rapid corrosion rate of bare ZK60 still limits its clinical applications. Therefore, MAO coating was introduced to enhance the corrosion resistance and treated as an intermediate layer for further surface modification. The purpose of MAO treatment is not only in building a protective layer, but also forming a large amount of hydroxyl groups on the magnesium surface for GA grafting. The aim of this study is to examine the influence of immobilizing GA onto the MAO coating with regard to corrosion resistance and osteocompatibility. The surface properties and corrosion behavior were, thus, characterized by scanning electron microscopy (SEM), X-ray photoelectron spectroscopy (XPS), and electrochemical tests. Furthermore, to investigate the selective cell regulation of GA toward fibroblast and osteoblast-like cells, both viability and cell attachment tests were performed.

## 2. Results and Discussion

### 2.1. Effects of MAO and Phenolic Monolayer on Coating Morphology

**Figure 1:**
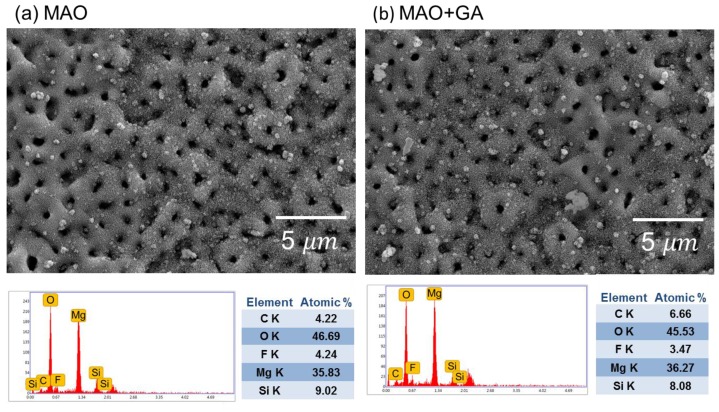
SEM images and EDX composition analysis of (**a**) MAO and (**b**) MAO+GA coating.

Figure 1 shows SEM images of the MAO- and MAO+GA-modified magnesium alloys’ morphologies. After MAO, the alloy surface became rough and coarse with numerous pores on it. The average diameter of the pores formed by the discharge channels of the MAO process was estimated at 1.1 µm. Since those pores were generated by the molten oxide and gas bubbles evaporating through the plasma, the pore size and thickness of the coating became bigger with the increase in oxidation time. Therefore, the random sparks of the MAO process caused the contours of the surface to have the shape of scattered volcanoes, with the resulting roughness of this ceramic coating, thus, having a larger surface area for grafting GA onto it. Most of the surface modification needed to be carried out in aqueous solution and, thus, the compactness of the protective coating might be destroyed. However, in this experiment, since GA could only be dissolved in absolute ethanol, the oxide layer would not react with water, break MAO coating’s structure, or generate cracks due to hydration and inflation. After the conjugation of GA, the morphology maintained its original structure because the GA layer was merely tens of nanometers thick.

### 2.2. The Coating Composition Analysis

**Figure 2:**
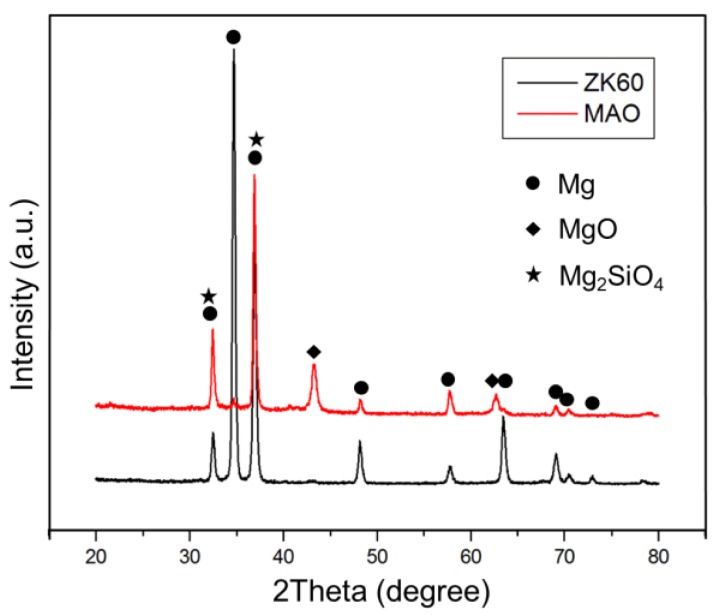
XRD patterns of ZK60 and MAO.

The EDX results (Figure 1) show that the composition of the MAO coating mainly consisted of O, Mg, F, Si, and C. The high oxygen content originated from the oxidized surface, with the high temperature plasma turning the magnesium into magnesium oxide on the sample’s surface. The fluorine ions in the electrolyte could react with the dissolved magnesium ions to form magnesium fluoride and so increase the compactness of the MAO layer and corrosion resistance [24]. However, the only difference between the MAO and MAO+GA groups was that the amount of carbon increased slightly from 4.22% to 6.66% due to the deposition of GA. The XRD patterns of the MAO indicated that the coating was composed of MgO (JCPDS 89-7746) and Mg_2_SiO_4_ (JCPDS 19-0768), which matched the EDX data and showed that the electrolyte participated in the MAO processing. (Figure 2) In addition, Mg_2_SiO_4_ could be decomposed into silicon, which is a trace element in human bone and can stimulate MG63 cell growth and support proliferation of osteoblast-like cells [14]. The peaks of Mg and Mg_2_SiO_4_ were partially overlapped at 32.2° and 36.8°, but the peak at 34.5° only belonged to Mg [25]. Therefore, the relative intensity of the peaks of Mg, MgO, and Mg_2_SiO_4_ indicated that the coating was successfully formed onto the substrate.

### 2.3. The Surface Chemical Bonding Characteristics

**Figure 3:**
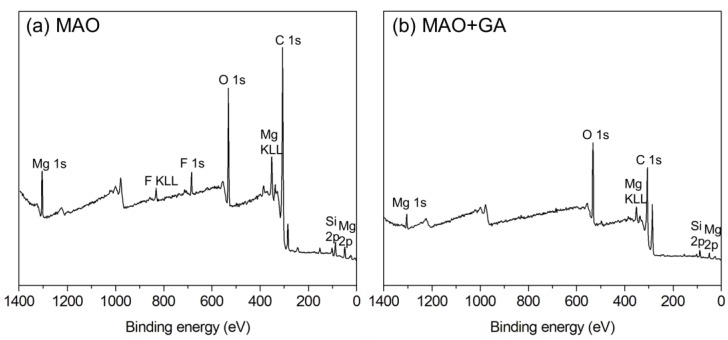
XPS spectra of (**a**) MAO and (**b**) MAO+GA coating.

**Figure 4:**
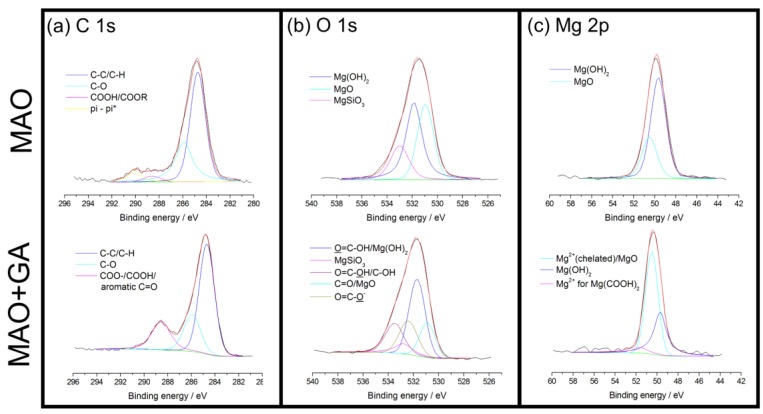
(**a**) C 1s; (**b**) O 1s, and (**c**) Mg 2p spectra of MAO and MAO+GA coating.

The GA complex layer merely occupied the outermost part of the coating, which can be detected by XPS. The atomic ratio agreed well with the EDS data, and showed that the content of all elements decreased except for that of carbon (Table 1). Due to the deposition of GA, the carbon content doubled from 26.6% to 55.3% and the rest of the elements in the MAO coating, especially Mg, Si, and F, were masked by the outer GA layer (Figure 3). To further investigate the bonding types of GA, high-resolution XPS spectra of C, O, and Mg elements were collected (Figure 4). According to the published data of XPS spectra for C 1s of MAO and MAO-GA, 284.7 eV and 285.9 eV were attributed to aliphatic C and C–O, respectively (Figure 4a) [26]. In addition, 288.6 eV was ascribed to COOH, COOR, COO^−^ and C=O (quinonyl) [27]. The area ratio of the carboxyl related group for the MAO-GA group, which is the characteristic of GA, was around 22%, proving that GA immobilizes on the MAO coating after the sample is immersed for 24 h. For O 1s, 530.9 eV belongs to aromatic C=O and magnesium oxide, while 531.7 eV is ascribed to O=C–OH and magnesium hydroxide (Figure 4b). Moreover, the peaks of magnesium silicate and O=C–O^−^ (magnesium carboxylate) are very close, which are 532.8 eV and 532.4 eV, respectively; 533.5 eV is assigned to the hydroxyl group and carboxyl group, and the targeted oxygen is different from the 531.7 eV peak of GA [27]. In the oxygen spectra of the MAO group, the data showed that the coating consisted mostly of magnesium oxide and magnesium silicate, which was agrees well with the EDX and XRD data. Furthermore, in the presence of water the outermost magnesium oxide might turn into magnesium hydroxide, with abundant sites for GA grafting. Therefore, the dehydration and condensation of GA and magnesium hydroxide led to formation of magnesium carboxylate [9]. A study showed that the carboxylic head group tends to dissociate hydrogen atoms, while the two oxygen atoms attach simultaneously to magnesium in the bidentate configuration with a stable binding mode [28]. For Mg 2p, the peak of magnesium hydroxide is at 49.7 eV. The peak at 50.5 eV can be attributed to chelated magnesium and magnesium oxide (Figure 4c); 51.6 eV is the magnesium ions, which belongs to magnesium carboxylate [27,28]. In the magnesium spectra, after the modification of GA, the magnesium hydroxide was replaced by chelated magnesium ions as the primary peak, which meant that magnesium hydroxide underwent the condensation process and the GA complex layer preserved the source of magnesium ions [29]. These ions chelated with either the quinonyl or carboxyl groups of GA to create coordination compounds. Since the stability of the gallic complex was usually high, those coordination compounds aggregated together into a steady multilayer structure [30,31]. Moreover, the electrostatic property of the gallic complex may reduce the penetration of the corrosive ions, especially chloride ions. In conclusion, these results confirm that GA firmly attached to the MAO ceramic coating through a stable binding mode.

### 2.4. The Electrochemical Tests

**Figure 5:**
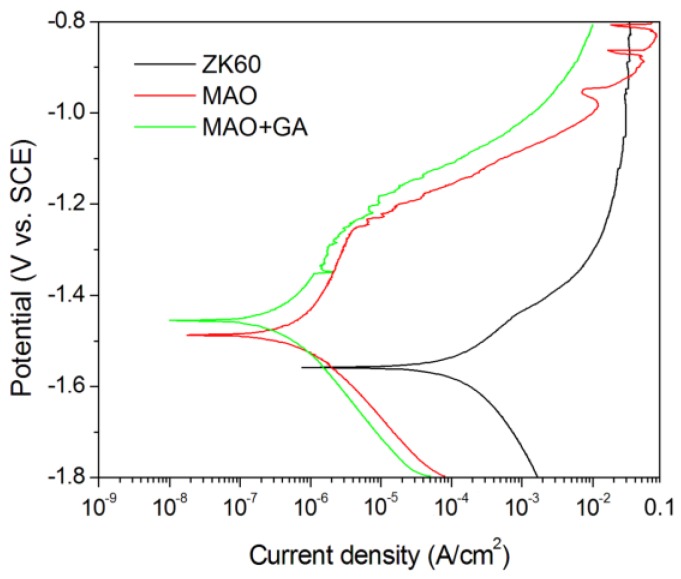
Potentiodynamic polarization curves of ZK60, MAO, and MAO+GA in SBF solution.

The corrosion parameter can be obtained from the potential dynamic curves by carrying out electrochemical tests in simulated body fluid, and the Tafel method then used to calculate the corrosion current. Among the ZK60, MAO, and MAO+GA groups, the ZK60 unmodified group showed the lowest corrosion potential at −1.564 V and the highest corrosion current density (9.6025 μA/cm^2^), which indicates it might suffer severe corrosion in physiological conditions (Figure 5). After MAO processing, the protective film can inhibit the corrosion fluid from contacting and reacting with the metal surface, and suppress the release of the hydrogen bubbles and alkali ions. The MAO group had higher corrosion potential at −1.485 V and its corrosion current density reduced dramatically to 0.583 μA/cm^2^ in comparison to ZK60 (Table 2). Furthermore, in the MAO-GA group, the corrosion potential increased slightly to −1.453 V and the corrosion current density further reduced to 0.372 μA/cm^2^ (Table 2). The results, thus, showed that the oxidation coating passivated the metal surface and increased its corrosion resistance, which is ascribed to the formation of an oxidized magnesium barrier. However, the unstable current density at a potential above −1.0 V indicated the occurrence of repassivation and pitting corrosion, due to the MAO coating having many pores [32]. In contrast, for the MAO+GA sample, not only was its corrosion resistance enhanced, but also the phenomenon of pitting corrosion disappeared. This implies that GA built a barrier to prevent the chloride ions from invading, and captured the magnesium ions to form magnesium carboxylates to buffer against pitting corrosion [27]. Moreover, it was difficult to immobilize molecules onto the magnesium or the protective coating surface in aqueous solution because of the concurrent corrosion. However, in the absolute ethanol solution, the oxidized coating was intact after immobilization of GA and, thus, this method can prevent deterioration of the function of the original coating.

### 2.5. Effects of Phenolic Monolayer on Cell Viability

**Figure 6:**
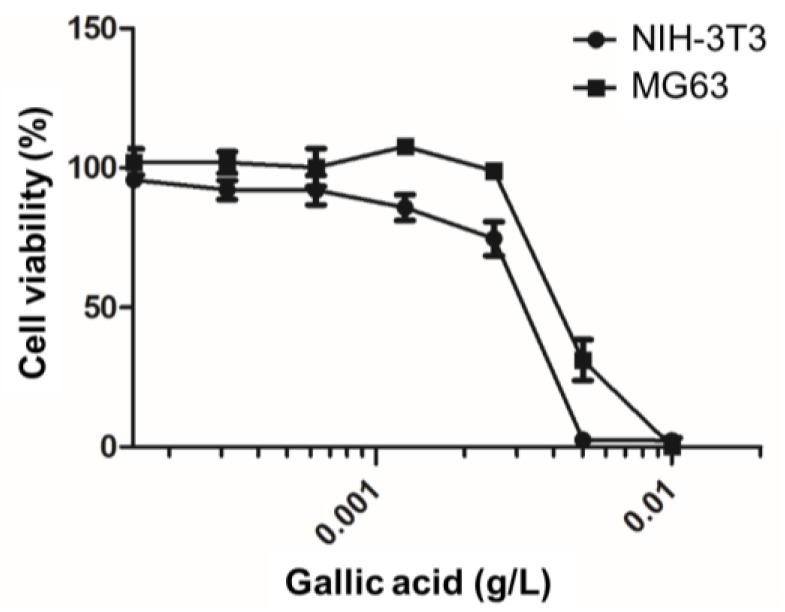
The viability of NIH-3T3 and MG63 co-cultured with varying gallic acid concentrations.

Many studies have focused on using GA to enhance endothelialization through the selective cell regulation between vascular endothelia (VEC) and vascular smooth muscle cells (VSMC) [33]. The function of selective cell regulation could also be applied to the bone-implant interface to inhibit fibrous capsule overgrowth and decrease inflammatory symptoms. Therefore, the response of the cell viability of MG63 and NIH-3T3 toward GA showed the selective response between bone-like cells and fibroblasts. The viability of MG63 was affected when the concentration of GA was above 0.0025 g/L (Figure 6). In contrast, NIH-3T3 was more vulnerable and the viability was only 73% for GA at 0.0025 g/L, and all the various concentrations of GA had little negative effects on the fibroblast cells. Moreover, it is worth noting that with a GA concentration ranging from 6.25 × 10^−4^ to 2.5 × 10^−3^ g/L, the viability was even better than seen with the lower concentration groups, which may imply that GA can stimulate MG63 growth in these particular conditions. At 1.25 × 10^−3^ g/L, it had the highest viability about 107% after 24 h co-culturing. Although GA did not show significant selective inhibition toward the two cell lines, it did suppress fibroblast growth and help bone-like cells proliferate.

### 2.6. The Cytocompatibility Evaluation

Cytocompatibility is the key parameter to evaluate whether implants have adverse effects on cells or accelerate the recovery of the surrounding tissue. Due to rapid corrosion, magnesium alloys may cause local alkalization, a high concentration of ions, and hydrogen evolution, which can generate an inflammatory situation. Moreover, the varying levels of inflammatory response and the thickness of the fibrous capsule imply the biocompatibility of the interface of the implants [34,35]. This study, thus, carried out an experiment to test the influence due to the release of the organic molecules of the GA-loaded interface on fibroblasts and osteoblast-like cells, respectively.

**Figure 7:**
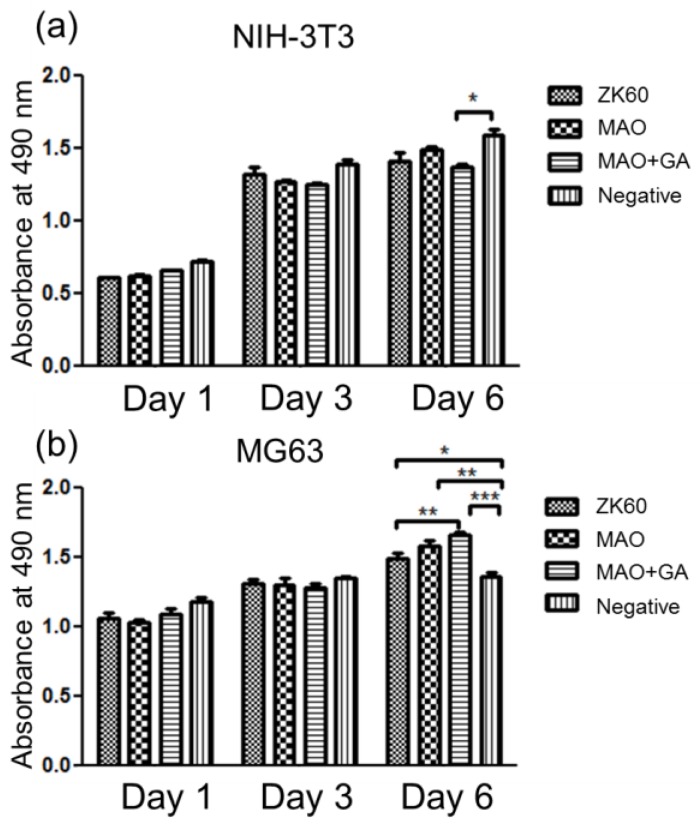
The cytocompatibility evaluation of ZK60, MAO, and MAO+GA with regard to (**a**) NIH-3T3 and (**b**) MG63. The data are presented as the mean ± SEM (*n* = 5). * *p* < 0.05, ** *p* < 0.01, *** *p* < 0.001.

In the MG63 test, all three groups, including ZK60, MAO, and MAO+GA, did not show any significant difference in cytocompatibility in days 1 and 3 (Figure 7b). An earlier study showed the corrosion rate of ZK60 was mild and that MG63 cells can tolerate the slightly increase in ion concentration [36]. Moreover, the magnesium ion was considered to be a beneficial factor which could stimulate the proliferation of osteoblasts in a mild environment. As such, the absorbance of all groups exceeded that of the negative groups on day 6. Due to the trace concentration of the released GA in the extracted medium, the selective stimulation seemed to be obvious, and more time was needed to accumulate the effects. Therefore, MAO+GA had greater cell viability than the other two groups until day 6, and the difference between the MAO+GA and negative group was significant, proving that the integrated effects of the magnesium ions and GA enhanced the proliferation of bone-like cells. In the NIH-3T3 test, the concentration of the released ion may exceed the tolerance of fibroblasts, and so the viabilities of all groups were lower than that of the negative group (Figure 7a). In addition, on day 6 MAO had the best performance in terms of viability, as the MAO coating controlled the corrosion behavior, so that the magnesium or alkaline ions did not burst out into the extracted medium. Furthermore, in contrast to MG63, GA showed inhibition of NIH-3T3 fibroblast cells, so MAO modified with GA had the lowest viability among all groups on days 3 and 6. The results indicated that although the concentration of GA was lower than the effective dose, the influence was preserved when the culturing was prolonged to day 6, similar to the results seen in the MG63 tests. The viability tests showed that the protective coating did not have a large difference for NIH-3T3 and MG63, as its concentration was in the tolerated limits of these two cell lines. Second, although the function of the selective cell inhibition of GA was not apparent on day 1, the effects could be accumulated with a longer culturing time. In conclusion, GA retained its influence after being immobilized on the oxidized compounds and, thus, stimulated bone-like cell proliferation and restrained the growth of fibroblasts.

### 2.7. The Cell Attachment Tests

The ability of the osteoblasts to attach on the implants is crucial for osseointegration, which can increase the strength of the bone-implant interface and its biocompatibility [37]. Since the micro-environment at the magnesium surface was different from the extracted medium in the viability tests, the higher concentration of the alkaline and magnesium ions, which were diluted by the extraction, may suppress cell attachment, while the hydrogen bubbles may reduce the space available for cell adhesion. According to numerous reports, the morphology and roughness of a surface could alter the biological response of osteoblasts and fibroblasts [38,39]. For example, with a smooth surface the fibroblasts tend to appear in a flattened and well-spread morphology, with decreased proliferation. Moreover, certain chemical groups, such as the carboxyl and hydroxyl groups of GA, could lead to better cell adhesion behavior through non-receptor routes, like hydrogen binding, and electrostatic and ionic-polar interaction [9]. Therefore, introducing GA onto a porous ceramic coating could affect cell adhesion by both physical and chemical interactions.

**Figure 8:**
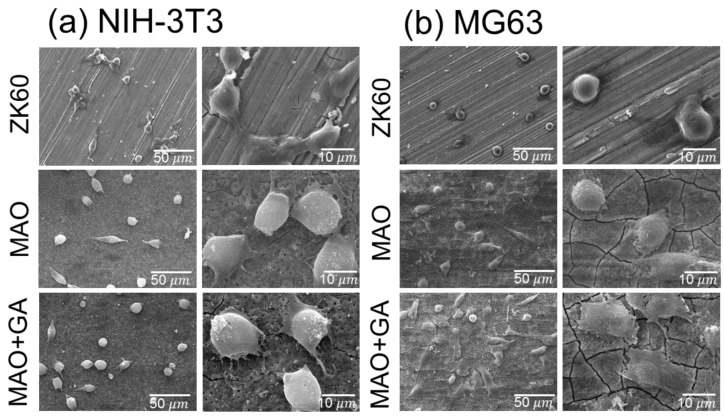
SEM images of (**a**) NIH-3T3 and (**b**) MG63 cells attachment on ZK60, MAO, and MAO+GA.

In the NIH-3T3 test, the unmodified ZK60 showed the lowest cell number, due to the adverse growing environment (Figure 8a). However, the NIH-3T3 adhered to the smooth surface seemed to have better attachment compared with that on MAO and MAO+GA, whereas on the rough surface the fibroblasts tended to only spread with occasional thin actin filaments, and remained in a round shape. Moreover, the attachment strength of fibroblasts on the MAO modified with a GA surface seemed to be fragile, in that some of the fibroblasts did not fully contact the surface and extended their pseudopodia. One study found that titanium modified with pyrogallol showed no obvious cytotoxicity to mammalian cells, but the surface resisted the attachment of NIH-3T3 fibroblasts and bacteria [17]. This result was in good agreement with other studies which found that fibroblast cells displayed less adhesive behavior on MAO+GA coating due to the rough and porous morphology, and the biological immobilization of GA.

**Figure 9:**
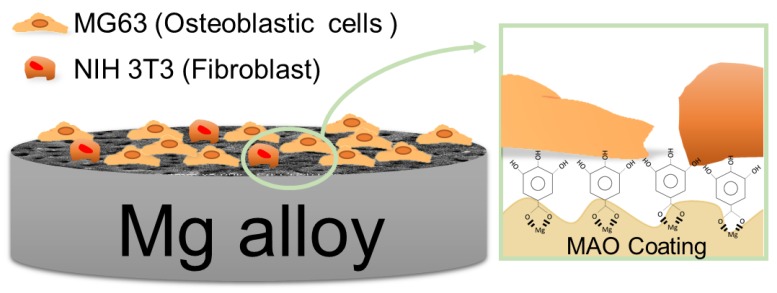
Schematic diagram of the MAO coating modified by gallic acid.

On the other hand, in the MG63 tests, the cell morphology of the adhered MG63 was completely different from that of the fibroblasts, due to the special characteristic of the osteoblast and osteosarcoma cells (Figure 8b). With regard to comparing the MAO and ZK60 groups, the morphology of the MG63 cells on the naked magnesium was in a spherical shape, without the extended pseudopodia, which indicated the cells did not adhere well. Second, the porous and coarse surface could increase the surface area for cell attachment, and was also suitable for osteoblast-like cell contact and proliferation [40]. Therefore, the morphology of cell attachment was notably improved for the MAO and MAO+GA groups. Third, in agreement with the results for NIH-3T3, the adverse effects of the severe corrosion were eliminated by the MAO coating, and MG63 cells more easily adhered on the MAO+GA than on ZK60. Due to the opposite response of fibroblast cells toward GA, for which the MG63 cells did not suffer from any suppression in the viability tests, the cell morphology showed good attachment for MAO+GA. In addition to the selective cell regulation, this result implied that the GA combined with the MAO coating on ZK60 also had a selective attachment toward the two different cell lines (Figure 9).

Previous studies indicated that the oxidative stress induced by reactive oxygen species (ROS) overproduction decreased the activity of osteoblasts [21]. In addition, ROS may indirectly control the cell adhesive and anti-apoptotic related proteins. Chen et al. [41] conjugated 3,4-dihydroxyhydrocinnamic acid onto chitosan to enhance antioxidant activity, and the results showed that the catechol-modified titanium surface could promote osteoblast adhesion and proliferation. GA also showed its radical scavenging ability as a basic phenolic compound after being grafted on bioactive ceramic [17,42]. GA might stimulate cell attachment through the elimination of ROS around the implant’s surface [43,44]. Moreover, the drilling procedure of bone grafting could generate numerous radicals around the interface between the bone and implant. Utilizing phenolic compounds to modify a bone graft has been shown to be an indirect, alternative method to accelerate bone healing in recent years. The surface properties of the implants have a significant influence on the biological response, and the absence of bioactive or stimulating factors (i.e., being inert) may lead to a variable thickness of fibrous tissue ,which is mainly composed of fibroblast cells [45]. Although forming fibrous capsules is unavoidable in order to reduce the inflammatory reaction, it also hinders osseointegration and nutrient supply. GA has been confirmed to be an anti-inflammatory compound, and the viability tests carried out in this study also showed that it preferentially suppressed fibroblast growth and attachment, which may reduce the thickness of fibrous capsules in vivo. Moreover, GA and MAO coating might promote the direct bone apposition ability of the orthopedic implants by stimulating proliferation and adhesion behavior, which provide long-term stability for the host and implant interaction. Additionally, GA can be a molecule for anchoring more diverse biomolecules, such as VEGF by co-immobilization, in future research [46].

## 3. Materials and Methods

### 3.1. Specimen Preparation

A commercial ZK60 alloy for use as a substrate was cut from alloy bars into cylindrical specimens with a diameter of 12.8 mm and height of 4 mm. The specimens were put into a preheated 400 °C furnace for 4 h to homogenize the composition and grain size. Those specimens were then ground with from #150 to #5000 SiC paper, ultrasonically cleaned in ethanol and de-ionized water for 5 min, and dried in a stream of air, and were then named the ZK60 group.

The MAO electrolytes were composed of 12 g/L Na_2_SiO_3_, 5 g/L NaOH, and 6 g/L NaF prepared with deionized water. The device for the MAO process consisted of a DC power supply, electrolytic bath, and cooling system. The ZK60 was processed at static current mode with 0.02 A/cm^2^ and the duration was 5 min, and this was name the MAO group. After rinsing the MAO-treated ZK60, the samples were immersed in 5 g/L gallic acid ethanol solution for 24 h, and then rinsed with distilled water and ethanol, and these were named the MAO+GA group.

### 3.2. Characterization of the Surface Properties

The surface morphologies and elemental composition of the MAO coating were studied by scanning electron microscopy and energy dispersed spectrometry (SEM/EDS). The crystallinity of the MAO coating was analyzed by thin film X-ray diffraction with Cu-Kα radiation (TF-XRD). Diffraction patterns were obtained between 2θ values of 20°–80°. The surface element configurations were determined by X-ray photoelectron spectroscopy (XPS).

### 3.3. Electrochemical Test

The tests were performed in revised simulated body fluid (r-SBF) solution (per liter, dissolved 5.403 g of NaCl, 0.736 g of NaHCO_3_, 2.036 g of Na_2_CO_3_, 0.225 g of KCl, 0.182 g of K_2_HPO_4_, 0.310 g of MgCl_2_·6H_2_O, 11.928 g of 4-(2-hydroxyethyl)-1-piperazineethanesulfonic acid (HEPES), 0.293 g of CaCl_2_, and 0.072 g of Na_2_SO_4_ in deionized water) [3]. A conventional three-electrode electrochemical cell was used for the polarization test with a platinum counter electrode and a saturated calomel electrode (SCE, +0.242 V vs. SHE) as the reference electrode. The r-SBF solution was then buffered to pH 7.4 at 37 °C by adding HEPES and NaOH at 37 °C by using a water bath. The polarization test used a PARSTAT 2273 electrochemistry workstation with a scanning rate of 1 mV·s^−1^ from −1.8 V to −0.8 V. The exposed area of the working electrode to the electrolyte was controlled by a Teflon holder to remain within 1 cm^2^.

### 3.4. Cell Culture and the Cell Viability Test

Human osteoblast-like cells (MG63, ATCC CRL-1427, Union Biomed Inc., Taipei, Taiwan) and mouse embryonic fibroblast cell line (NIH-3T3, ATCC CRL-1658, Union Biomed Inc., Taipei, Taiwan) were cultured in Dulbecco’s Modified Eagle’s Medium (DMEM, Gibco, Thermo Fisher Scientific Inc., Waltham, MA, USA) with high glucose, supplemented with 10% fetal bovine serum (FBS), and 1% antibiotics. These materials can stimulate the growth of osteoblasts and inhibit the proliferation of fibroblasts and, thus, provide good osteointegration between materials and bone. The selective cytotoxic properties of GA were evaluated by using a CellTiter-96 assay. The two kinds of cells were seeded onto 96-well plates with 2000 cells per well respectively, and cultured for 24 h for cell adhesion prior to the addition of the extractant from all groups. GA was diluted into various concentrations in DMEM, and then 100 μL in solution was added to each well. After the cells were incubated for 24 h, the medium was replaced with 100 μL medium with 10 μL of CellTiter-96 solution, and moved into an incubator for 4 h. The negative control group used culture medium, while the positive control group used culture medium with 5% dimethyl sulfoxide (DMSO). The absorbance of the CellTiter-96-treated solution was measured using an enzyme-linked immunosorbent assay (ELISA) reader at a wavelength of 490 nm. The viability was calculated according to the following formula:

Viability = [(OD_test_ − OD_blank_)/(OD_negative_ − OD_blank_)] × 100%

The cytotoxicity tests of the samples were also evaluated by using a CellTiter-96 assay and an indirect method. The samples, ZK60, MAO, and MAO+GA, were immersed into DMEM medium for 24 h to obtain extracts. MG63 and NIH-3T3 cells were separately cultured in 96-well plates at 5 × 10^3^ cells/100 μL for 24 h to allow for initial cell adhesion with normal medium. The medium was then replaced with 100 μL extract. After the cells were incubated for one, three, and six days, CellTiter-96 assay was applied to obtain the absorbance using the same method as above.

### 3.5. Cell Adhesion Tests

NIH-3T3 and MG63 were also used to investigate the earlier cell adhesion behavior on the ZK60, MAO, MAO+GA surfaces. The specimens were sterilized using ethanol before cell seeding. Cells (about 5 × 10^3^) were seeded on the surface in each well and cultured at 37 °C with 5% CO_2_ for 24 h. For cell observation, cells on samples were fixed with 4% paraformaldehyde solution and rinsed three times with phosphate-buffered solution (PBS, pH 7.4). The specimens were then dehydrated in 30 vol %, 50 vol %, 70 vol%, 90 vol %, 95 vol %, 100% alcohol, and hexamethyldisilazane (HMDS) solutions. Finally, the samples were sputter-coated with gold for SEM observations.

## 4. Conclusions

This research successfully conjugated GA onto MAO ceramic coating through reactions between the hydroxyl group of magnesium hydroxide and carboxyl group of GA. The MAO coating also enhanced the corrosion resistance and the biocompatibility of the magnesium substrate. The GA thin film was mainly composed of magnesium ions and GA, which chelated together to form a relatively stable structure. In vitro tests showed that GA has selective regulation toward fibroblasts and osteoblast-like cells, which implies that it can suppress fibroblast growth and stimulate bone-like cells proliferation, simultaneously. Further, since the MAO+GA coating was porous and osteocompatible, MG63 finds it facile to attach on the magnesium substrate. Therefore, through the use of an appropriate post-treatment, magnesium alloys have the potential to be an applicable biomaterial with suitable degradation duration and osteointegration.

## Figures and Tables

**Table 1:** XPS results of elemental concentration.

Group	Element concentration (at.%)				
	O	C	Mg	Si	F
MAO	42.6	26.6	19	6.2	5.6
MAO+GA	33.3	55.3	8.2	1.9	1.3

**Table 2:** Fitting results of potentiodynamic polarization curves related to Figure 5.

Group	E_corr_ (V)	I_corr_ (μA)
ZK60	−1.564	96.025
MAO	−1.485	0.583
MAO+GA	−1.453	0.372
